# The Literature of the Times

**Published:** 1861-08

**Authors:** 


### THE LITERATURE OF THE TIMES.

In the earliest ages the mode of imparting information was verbal; was by familiar communications. Later, Homer sung and philosophers recited in more set forms. Further on in time, the steel on the stone; then the pen on papyrus; next, books; last, newspapers and the periodical press; this is an agency which stretches its arm over the habitable globe; no deep ravine, no mountain top; nor broad savannas, nor shoreless seas; no prairie, no forest which it does not reach; it penetrates the prisoner's dungeon; it reaches to the courts of kings; not a page that does not make its mark, not a line but has its influence; and whether for ill unending, or eternal good, depends on the character of that line or page, its falsehood or its truth; one or the other it must be.

The periodical press is now the chief educator of the masses of civilized lands, and it is always at work; in the silence of night as well as in the glare of day; in the hum of the city as well as in the secret closet; and most, may-be, in the darkness and in the closet, for then and there reflection comes, aided by stillness and silence, to deepen impressions and fix ideas in the brain, ready for the action of the life.

Water falling on the head drop by drop on the same unchanged spot, makes a man a raving maniac in very brief space; so ideas, one by one, falling on the brain or impressing the heart, have an influence for the terrible or the blessed not less certain than the ceaseless falling drops, to craze that brain, to crush out that heart's affections, or to lighten, beatify, and bless.

To supervise then the ideas, the reading which comes day by day in their tiny instalments of drop by drop upon the brains and hearts of the young, would seem to be among the first things of a father's duty, of a mother's care. And if in this age of bustle and hurry, parents and guardians have not the time to read first all that comes before the eyes of the young, the next best course is to purchase such books and patronize such publications as come from men whose past lives and writings give assurance that nothing can come from them which is not pure, profitable, and true; discarding resolutely that large class of publications which panders to the prevailing opinions of to-day, which at one time is virtuous, at another vicious; at one time on the side of law and order, at another standing shoulder to shoulder with disorganizers; at one time for the Sabbath, at another against it; at one time for the Bible and for religion, but ready at any hour, for a consideration, to lend temporary aid and comfort to the enemies of both. Let all the good, then, have to do with those who, in a true sense, are the same all the time.

